# Optically Induced Static Magnetization in Metal Halide Perovskite for Spin‐Related Optoelectronics

**DOI:** 10.1002/advs.202004488

**Published:** 2021-05-02

**Authors:** Miaosheng Wang, Hengxing Xu, Ting Wu, Haile Ambaye, Jiajun Qin, Jong Keum, Ilia N. Ivanov, Valeria Lauter, Bin Hu

**Affiliations:** ^1^ Joint Institute for Advanced Materials Department of Materials Science and Engineering University of Tennessee Knoxville TN 37996 USA; ^2^ Neutron Scattering Division Neutron Sciences Directorate Oak Ridge National Laboratory Oak Ridge TN 37831 USA; ^3^ Center for Nanophase Materials Science and Chemical and Engineering Materials Division Oak Ridge National Laboratory Oak Ridge TN 37831 USA; ^4^ Chemical and Engineering Materials Division Oak Ridge National Laboratory Oak Ridge TN 37831 USA

**Keywords:** heterojunction, hybrid perovskite, photoinduced magnetization, polarized neutron reflectometry, spintronics

## Abstract

Understanding the feasibility to couple semiconducting and magnetic properties in metal halide perovskites through interface design opens new opportunities for creating the next generation spin‐related optoelectronics. In this work, a fundamentally new phenomenon of optically induced magnetization achieved by coupling photoexcited orbital magnetic dipoles with magnetic spins at perovskite/ferromagnetic interface is discovered. The depth‐sensitive polarized neutron reflectometry combined with in situ photoexcitation setup, constitutes key evidence of this novel effect. It is demonstrated that a circularly polarized photoexcitation induces a stable magnetization signal within the depth up to 7.5 nm into the surface of high‐quality perovskite (MAPbBr_3_) film underneath a ferromagnetic cobalt layer at room temperature. In contrast, a linearly polarized light does not induce any detectable magnetization in the MAPbBr_3_. The observation reveals that photoexcited orbital magnetic dipoles at the surface of perovskite are coupled with the spins of the ferromagnetic atoms at the interface, leading to an optically induced magnetization within the perovskite’s surface. The finding demonstrates that perovskite semiconductor can be bridged with magnetism through optically controllable method at room temperature in this heterojunction design. This provides the new concept of utilizing spin and orbital degrees of freedom in new‐generation spin‐related optoelectronic devices.

## Graphical abstract

The depth‐sensitive polarized neutron reflectometry technique reveals the optically induced static magnetization in metal halide perovskite by coupling photoexcited orbital magnetic dipoles with spins at perovskite/ferromagnetic interface. This work demonstrates that perovskite can be bridged with magnetism through optically controllable method in perovskite/ferromagnetic heterojunction, providing the new concept of utilizing spin and orbital degrees of freedom for spin‐related optoelectronics.

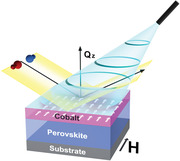

## 1 Introduction

The metal halide perovskites have attracted intense research efforts for developing solution‐processed thin‐film photovoltaic and light‐emitting devices.^[^ ^1^, ^2^ ^]^ Recently, the metal halide perovskites have demonstrated three intriguing spin‐related phenomena: spin injection,^[^ ^3^, ^4^, ^5^, ^6^ ^]^ electric–magnetic coupling,^[^ ^7^ ^]^ and optically generated spin states.^[^ ^8^, ^9^ ^]^ The success of spin injection provides a precondition to realize the spin‐dependent charge transport and excited states in metal halide perovskites by using externally introduced spins even with the presence of strong spin‐orbit coupling (SOC). Moreover, the observed electric–magnetic coupling at the perovskite/Co interface in dark condition presents fundamental feasibility to couple semiconducting and magnetic properties through interface design. The optically generated spin states were observed in a variety of metal halide perovskites at room temperature.^[^ ^10^, ^11^, ^12^ ^]^ This shows that when circularly polarized photoexcitation is used to generate excitons with the same‐directional orbital magnetic dipoles, the interaction between orbital magnetic dipoles can be established between excitons at the condition that spin polarizations are conserved within exciton lifetime. These phenomena present a fundamental question: do optically photoexcited spin states have sufficiently long lifetime to interact with ferromagnetic surface spins to generate optically induced magnetization? Photoinduced magnetization was demonstrated in plasmonic nanoparticles via inverse Faraday effect by transferring the angular momentum from the optical field to the electron gas.^[^ ^13^ ^]^ However, static photoinduced magnetization is generally difficult to realize in conventional semiconductors due to the short spin lifetime as compared with the long carrier lifetime.^[^ ^14^ ^]^ Recently, a long spin relaxation time of up to ≈190 ps was observed in perovskite (MAPbBr_3_) at room temperature and was attributed to the presence of surface Rashba state.^[^ ^12^ ^]^ Meanwhile, we observed that switching the photoexcitation between linear and circular polarization leads to a change in photoluminescence (PL) intensity, generating a ΔPL phenomenon in perovskites at room temperature.^[^ ^15^, ^16^ ^]^ This observation indicates that the orbit–orbit interaction between excitons can indeed occur in the time window of the PL lifetime, thus providing a promising opportunity for developing optically induced magnetization using optically generated spin states that interact with the ferromagnetic surface.

In this work, we present experimental evidence of this new phenomenon: the capability to optically induce magnetization by combining photoexcited spin states on the surface of perovskite film and the magnetic dipoles on the ferromagnetic Co interface with perovskite layer. The well‐defined MAPbBr_3_/Co interface was prepared by thermally depositing a Co layer on the surface of the ultrasmooth perovskite layer spin‐casted on a Si wafer. The magnetodielectric measurements demonstrate that the MAPbBr_3_/Co interface exhibits an electric–magnetic coupling, through which the spin on the ferromagnetic Co surface and the orbital field on the perovskite surface can be coupled for spin manipulations. Notably, by using the depth‐sensitive polarized neutron reflectometry (PNR) combined with an in situ circularly polarized laser beam (488 nm) excitation, a clear magnetization with a thickness of several nanometers was observed in the MAPbBr_3_ layer near the MAPbBr_3_/Co interface. This photoexcitation‐induced magnetization disappears when switching the photoexcitation from circular to linear polarization, similar to dark condition. Our study demonstrates that circularly polarized orbital magnetic dipoles in metal halide perovskites can be magnetically aligned by the spin dipoles of ferromagnetic Co surface through the perovskite/ferromagnet interface, functioning as optically induced ferromagnetic states within the perovskite surface. Thus, in this heterojunction design, perovskite semiconductor can be bridged with magnetism through optically controllable method at room temperature, which provides the new concept of utilizing spin and orbital degrees of freedom in new‐generation semiconductor devices, including spin field‐effect transistor, ultradense nonvolatile semiconductor memory, and spin LEDs.^[^ ^17^ ^]^

## 2 Results

**Figure 1:**
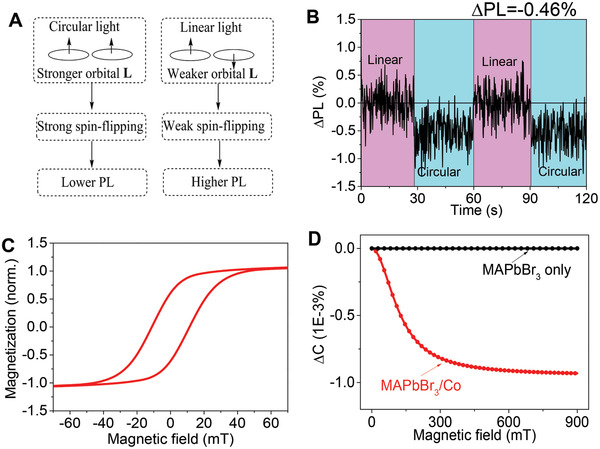
Photoluminescence (PL) and magnetic properties. A) Schematic diagram representing the generation of polarization‐modulated photoluminescence in metal halide perovskites by switching photoexcitation polarization between circular and linear polarization. B) PL change caused by switching the photoexcitation from linear to circular. The orbital magnetic dipoles are formed in spin‐polarized excited states under circularly polarized photoexcitation. C) Magnetic hysteresis loop of the MAPbBr_3_/Co/Au sample. D, Magnetodielectric effects from the two different device configurations: Indium tin oxide (ITO)/polymethyl methacrylate (PMMA)/MAPbBr_3_/Co/PMMA/Ag; ITO/PMMA/MAPbBr_3_/PMMA/Ag with the frequency of 1 kHz at dark condition.

The polycrystalline MAPbBr_3_ thin films were prepared using the solution spin‐casting method. The absorption and PL spectra are shown in Figure S1A in the Supporting Information. A sharp absorption onset is observed at 543 nm and the PL emission peak is located at 537 nm with the full width at half maximum of 21 nm, which represents the typical semiconducting properties of MAPbBr_3_ thin films. The out‐of‐plane X‐ray diffraction pattern showing sharp (001) and (002) peaks confirms well‐ordered crystals of cubic MAPbBr_3_ with (001) orientation (Figure S1B, Supporting Information). The photoexcited orbital magnetic dipoles were identified in the MAPbBr_3_ thin film by monitoring PL intensity while switching the photoexcitation between linear and circular polarizations. Note, a circularly polarized photoexcitation generates same‐direction orbital magnetic dipoles in excited states before spin relaxation occurs (**Figure** **1**A). In contrast, a linearly polarized photoexcitation leads to opposite‐direction orbital magnetic dipoles. The orbital magnetic dipoles generated by circularly polarized photoexcitation on the perovskite surface provide the necessary conditions for interaction with magnetic spins on the ferromagnetic surface. Figure 1B shows the PL intensities measured with linearly and circularly polarized photoexcitations at the same intensity. We see that circular and linear photoexcitations generate lower and higher PL intensities respectively, leading to a ΔPL phenomenon. By monitoring the change of PL intensity with switching the photoexcitation polarization, the left‐hand and right‐hand circularly polarized light excitations give the same PL intensity (Figure S2, Supporting Information). The ΔPL phenomenon is fundamentally different from circular dichroism. Circular dichroism originates from the differential absorption of left‐hand and right‐hand circularly polarized light, generally observed in the chiral structure.^[^ ^18^ ^]^ Here, we should emphasize that, when orbit–orbit interaction occurs before spin relaxation, the same‐direction and opposite‐direction orbital magnetic dipoles generate stronger and weaker SOC between excited states, leading to increased and reduced efficiency of intersystem crossing from optically generated bright states to dark states with higher and lower PL intensities, respectively. This causes a ΔPL phenomenon upon switching photoexcitation between linear and circular polarizations when photoexcited orbital magnetic dipoles establish a mutual interaction within PL lifetime. Therefore, the ΔPL phenomenon provides a convenient method to identify whether orbital magnetic dipoles can develop a mutual interaction before spin relaxation in perovskites. This characterization serves as the preconditions to enable the photoinduced magnetization in perovskite in this work.

To investigate the perovskite/ferromagnetic interface, we engineered a double‐layered heterostructure by depositing a 20 nm Co layer on top of the MAPbBr_3_ perovskite layer using the thermal evaporation method. Then the 15 nm Au layer was subsequently grown to protect the Co layer from oxidation. The X‐ray reflectivity (XRR) curve of this sample indicates the well‐controlled interfacial roughness around 1 nm over the whole sample (2 × 2 cm^2^), which is suitable for reflectometry measurements (Figure S3, Supporting Information). The fit parameters are shown in Table S1 in the Supporting Information. The 20 nm Co layer forms a continuous polycrystalline film with typical ferromagnetic properties. The MAPbBr_3_/Co/Au sample shows a clear magnetic hysteresis loop in the in‐plane direction, as shown in Figure 1C. Here, we used the magnetodielectric measurement to investigate the MAPbBr_3_/Co interface. Figure 1D illustrates that the capacitance signal measured at frequency of 1 kHz gradually decreases with the increased applied magnetic field, leading to a magnetodielectric signal from the MAPbBr_3_/Co interface. In contrast, when the Co layer is absent (MAPbBr_3_‐only device), negligible magnetodielectric signal was detected. The observed magnetodielectric signal in the MAPbBr_3_/Co sample originates from the electric‐magnetic coupling between the spins of the ferromagnetic Co atoms and electric polarization at the perovskite surface.^[^ ^7^ ^]^

**Figure 2:**
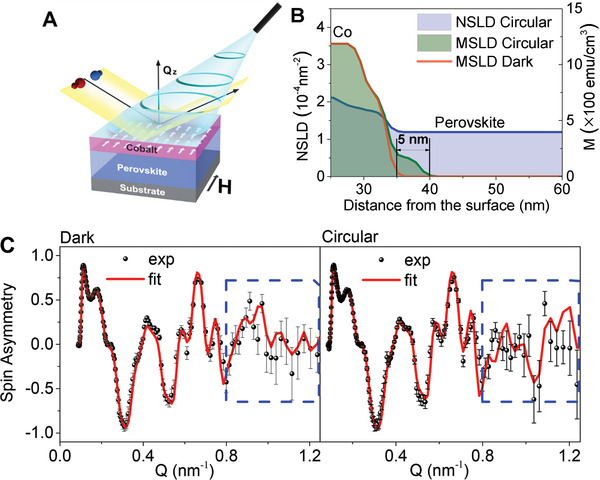
Results from probing the interface magnetism using polarized neutron reflectometry (PNR) experiment with in situ photoexcitation. A) Schematic of the experiment on an MAPbBr_3_ perovskite/Co structure. The external magnetic field *H* is applied in‐plane to the sample. The polarized neutron beam with neutrons spins parallel (blue arrow) or opposite (red arrow) impinging under the grazing angle at the sample. The 488 nm laser beam illuminates the entire surface of the sample (see Figure S6, Supporting Information). The neutron beam probes the sample in dark and under circularly polarized photoexcitation conditions. B) The perovskite/Co interface region represented as a plot of magnetic layer thickness as a function of the distance from the surface, obtained from fitting the specular PNR reflectivity (see Figure S4A, Supporting Information). Overall, the magnetization in a 5 nm MAPbBr_3_ perovskite layer (black lines) is observed under the circularly polarized laser beam excitation (green). In contrast, this magnetization effect is absent under dark condition (red solid line). The chemical composition of the interface (NSLD), shown in light purple, confirms that no Co atoms are present in the perovskite. The interfacial roughness determined from the PNR experiment is 0.5 nm. C) The spin asymmetry ratio, SA = (*R*+ − *R−*)/(*R*+ + *R−*), obtained from the experimental and fitted reflectivities is shown for measurements performed under dark condition (left) and circularly polarized laser beam excitation (right). The blue dashed box highlights the difference in SA between the dark and circularly polarized laser beam excitation conditions in the high‐Q range, due to differences in magnetization profile at the perovskite/Co interface. The error bars represent one standard deviation.

The electric–magnetic coupling at the MAPbBr_3_/Co interface raises an important question about whether the ferromagnetic Co layer can magnetically interact with photoexcited orbital magnetic dipoles in the perovskite layer, introducing optically induced magnetization on the MAPbBr_3_ surface. To explore optically induced magnetism at the MAPbBr_3_/Co interface, we used the PNR technique combined with the experimental setup for in situ photoexcitation. PNR is a highly penetrating depth‐sensitive technique to probe the chemical and magnetic depth profiles with a resolution of 0.5 nm. The depth profiles of the nuclear and magnetic scattering length densities (NSLD and MSLD) correspond to the depth profile of the chemical and in‐plane magnetization vector distributions on the atomic scale, respectively.^[^ ^19^, ^20^, ^21^ ^]^ Based on these neutron scattering merits, PNR serves as the powerful technique to simultaneously and nondestructively characterize chemical and magnetic nature of buried interfaces.^[^ ^22^ ^]^ Magnetism arises from the strong short‐range correlation between electronic spin and orbital degree of freedom, which can be inherently altered at interfaces, particularly in the presence of strong SOC and external stimuli.^[^ ^23^ ^]^ Upon in situ photoexcitation during the PNR measurements (**Figure** **2**A), the change of magnetization properties at MAPbBr_3_/Co interface can be detected by monitoring the MSLD depth profile. Here, the PNR measurements were completed first on the Si/MAPbBr_3_/Co/Au sample (N1) at room temperature, and in‐plane applied magnetic field of 1 T. The neutron reflectivity plots of R^+^ and R^−^, where the superscript plus (or minus) signs indicate neutrons with spin parallel (or antiparallel) to the direction of the applied magnetic field, are shown in Figure S4A in the Supporting Information as a function of the wave vector transfer *Q* = 4*π*sin(*θ*_i_/*λ*), where *θ*_i_ is the incident angle and *λ* is the neutron wavelength. The chemical profile (NSLD) and the magnetic profile (MSLD) are obtained from a simultaneous fit of the PNR data (Figure S4B, Supporting Information) and are plotted as functions of the distance from the sample surface. By closely comparing the MSLD_dark_ with the NSLD in Figure 2B with the focus on the perovskite/Co interface region, we can see that in dark condition, the magnetism is concentrated solely in the ferromagnetic Co layer. However, when under circularly polarized 488 nm continuous‐wave laser beam photoexcites orbital magnetic dipoles to generate spin‐polarized excited states, the difference between R+ and R− curves becomes larger in the high Q range from 0.8 to 1.2 nm^−1^, compared to dark condition measurements, indicating the appearance of interfacial magnetization induced by circularly polarized photoexcitation within the perovskite layer near the perovskite/ferromagnetic interface. The corresponding spin asymmetry of the circularly polarized photoexcitation condition also shows a larger value in the high Q range compared to the dark condition, representing the enhanced magnetization within the perovskite interface (Figure 2C). Indeed, the corresponding MSLD_circular_ depth profile shows that the magnetization reaches into the perovskite layer ≈5 nm at the MAPbBr_3_/Co interface under circularly polarized laser excitation. Note, this additional magnetization does not come from the Co atoms because the NSLD profile indicates the pristine MAPbBr_3_ component in the same depth regime, which was confirmed using XRR (Figure S3, Supporting Information).

**Figure 3:**
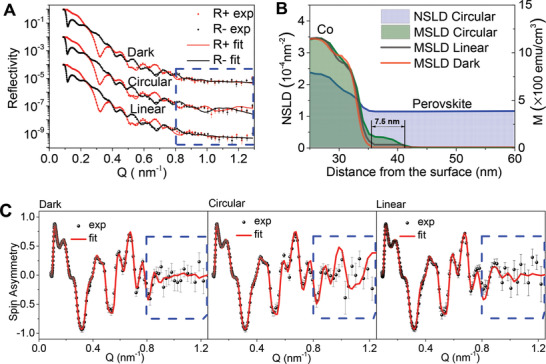
PNR experiments under dark, circularly, and linearly polarized photoexcitation. A) Polarized neutron reflectivity plots for the second MAPbBr_3_ perovskite/Co sample (N2) measured in the dark (top), under circularly polarized photoexcitation, and linearly polarized photoexcitation (bottom). The blue dashed box highlights the difference between dark, circularly, and linearly polarized laser beam excitation conditions in high‐Q range. B) Chemical (NSLD) and magnetization (MSLD) profiles in these three measurement conditions obtained from the fit to the reflectivity curves with the focus on the perovskite/Co interface region. C) Spin asymmetry plots under dark (left), circularly (middle), and linearly (right) polarized laser beam excitation. The blue dashed box highlights the difference between dark, circularly, and linearly polarized laser beam excitation conditions in the high‐Q range. The error bars represent one standard deviation.

To confirm the observed photoinduced magnetization at the MAPbBr_3_/Co interface, we performed a detailed study on a second Si/MAPbBr_3_/Co/Au sample (N2) with a similar structure to explore magnetization under dark, circularly and linearly polarized photoexcitation (**Figure** **3**A). Figure 3B depicts the chemical (NSLD) and magnetization (MSLD) profiles for dark, linear, and circular laser polarizations at the perovskite/Co interfacial region, and the complete NSLD and MSLD profiles are shown in Figure S5 in the Supporting Information. As illustrated in Figure 3B, no magnetization was detected in the MAPbBr_3_ layer at the MAPbBr_3_/Co interface in dark condition. However, when the circularly polarized 488 nm laser beam excited the MAPbBr_3_, we observed that magnetization formed in MAPbBr_3_ layer with the thickness of 7.5 nm at MAPbBr_3_/Co interface. This is consistent with the experimental results observed in sample N1. In contrast, when switching the photoexcitation to a linearly polarized laser beam, the interfacial magnetization is significantly reduced to a negligible value within the sensitivity of the method. Our results of the observed interface magnetization are intrinsically different from the charge carrier effect where the photogenerated charge carriers from MAPbI_3_ can decrease the magnetism of the ferromagnetic La_1−x_Sr_x_MnO_3_ (LSMO) in MAPbI_3_/LSMO heterojunction through the photodoping‐driven modulation of the Curie temperature (*T*_C_) of LSMO.^[^ ^24^ ^]^ Instead, the orbital magnetic dipoles in spin‐polarized states generated by circular photoexcitation are likely the origin of the observed photoinduced interface magnetization in MAPbBr_3_ layer. The reflectivity spin asymmetry plots in Figure 3C show that the circularly polarized photoexcitation condition yields a larger spin asymmetry value in the region of wavevector transfer (0.8 nm^−1^ < *Q* < 1.2 nm ^‐1^), which corresponds to the length scale of 6–8 nm, and the fit to the data shows that it comes from the interface magnetization extending for 7.5 ± 0.5 nm into MAPbBr_3_ from the interface. From the fit to the data, we obtained detailed quantitative information on the magnetization induced in the perovskite interfacial layer. The next to Co perovskite layer (sublayer 1) is 6.8 ± 0.5 nm wide with an MSLD = (3.4 ± 1) × 10^−5^ nm^−2^, which corresponds to 117 ± 30 emu cc^−1^. The next layer, sublayer 2, is about 0.7 ± 0.5 nm wide with an MSLD = (1.5 ± 0.5) × 10^−5^ nm^−2^, which corresponds to 51.7 ± 17 emu cc^−1^. The magnetization of the Co layer has a maximum MSLD Co = (3.34 ± 0.1) × 10^−4^ nm^−2^, corresponding to 1151.7 ± 30 emu cc^−1^ (see Figure 3B). In the PNR technique, it is important to use a large‐area thin film sample (2 × 2 cm^2^ in this work) to increase the data collection efficiency and to maximize the signal from the neutron beam. Thus, the laser beam was expanded to uniformly illuminate the entire sample surface, leading to limited excitation density of ≈ 14 mW cm^−2^. Under this reduced excitation density, the photoinduced magnetization in MAPbBr_3_/Co heterojunction was still successfully observed under circularly polarized excitation condition in sample N1 and confirmed in an additional sample N2.

**Figure 4:**
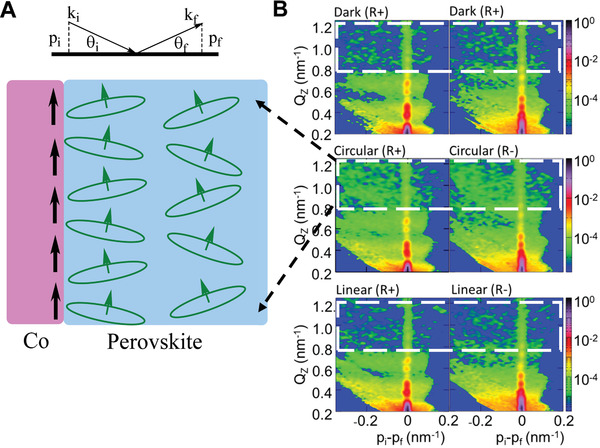
Diagram demonstrating optically induced magnetization. A) The orbital magnetic dipoles are coupled with circularly polarized photoexcitation generated excited states with magnetic spins at MAPbBr_3_/Co interfaces. B) R+ and R− experimental 2D maps of the intensity scattered from the MAPbBr_3_/Co sample as a function of *p*_i_ − *p*_f_ and *Q*z = *p*_i_ + *p*_f_, where *p*_i_ and *p*_f_ are perpendicular to the sample surface components of the incoming and the outgoing wave vectors, respectively (see inset in (A)). The logarithmic intensity color scale is shown on the side. The strong intensity along the vertical lines at *p*_i_ *– p*_f_ = 0 corresponds to the specular reflectivity, which have been extracted and analyzed in Figure 3A. The 2D map in the middle of the panel (B) was measured under circular polarization condition. The orbital magnetic dipoles result in additional magnetic scattering for *Q* > 0.8 nm^−1^, which is absent in the top (dark) and bottom (linear polarization) panels and marked with a white dashed rectangle.

Clearly, circularly polarized photoexcitation can introduce a magnetization at the MAPbBr_3_/Co interface, representing a promising method to realize magnetized excited states through the coupling between the orbital magnetic dipoles of perovskite and the spin dipoles of ferromagnetic surface. The observed ΔPL phenomenon provides evidence that the orbital magnetic dipoles can conserve their spin polarizations within the lifetime of excited states, providing the necessary condition to interact with the spin dipoles of ferromagnetic surface toward developing optically induced magnetization. **Figure** **4**A illustrates the principle of optically induced magnetization by directly coupling the orbital magnetic dipoles of circularly polarized excited states on the perovskite surface with the magnetic spins on the ferromagnetic Co surface. The coupling between orbital magnetic dipoles of excited states generated by circular photoexcitation and magnetic spins of Co is an underlying mechanism of the optically induced magnetization through the perovskite/ferromagnetic interface. To further confirm the mechanism, we measured the off‐specular scattering^[^ ^22^ ^]^ with polarized neutrons under the same experimental conditions with in situ circularly polarized, linearly polarized photoexcitations, and under dark conditions. Method of PNR is a 3D vector magnetometry technique with the depth resolution of 0.5 nm, which is sensitive to the direction and the value of the magnetic moment and the lateral dimensions of magnetic domains. The scattering process consists of specular reflection and off‐specular scattering. For polarized neutrons, each of these processes consists of four scattering channels: four nonspin‐flip and four spin‐flip. Depending on the physical processes which are happening in the system, scattering in different channels is determined by different parts of those processes and shows up differently (stronger or weaker, or absent) in the experiment. The measurement and analysis of those scattering channels allow to provide a complete picture of the effect. The magnetic off‐specular scattering has a spin‐flip origin^[^ ^25^ ^]^ and is sensitive to the lateral fluctuations of the magnetic moments.^[^ ^26^, ^27^ ^]^ The orbital magnetic dipoles of excited states generated by polarized photoexcitation are coupled with the magnetic spins at perovskite/ferromagnetic interfaces as shown in the scheme in Figure 4A. The neutron spins undergo spin‐flip scattering^[^ ^25^ ^]^ on the in‐plane component of the magnetic dipoles perpendicular to the neutron spin,^[^ ^28^, ^29^ ^]^ and this scattering appears as an additional intensity in the two‐dimensional (2D) intensity maps. The results of off‐specular scattering measurement for the three experimental conditions with circularly polarized, linearly polarized photoexcitation, and dark conditions are presented in Figure 4B as a 2D intensity map in the coordinates *p*_i_ − *p*_f_ and *p*_i_ + *p*_f_ (*p*_i_ and *p*_f_ are shown in the inset and are the components of the neutron wavevectors perpendicular to the sample surface). The *Q* = *p*_i_ + *p*_f_ is the wave vector transfer perpendicular to the surface, so the structure of the multilayer along this direction is probed. The intensity of the specular line as a function of *p*_i_ + *p*_f_ at *p*_i_ − *p*_f_ = 0 reflects the features described in the following. The total reflection region at low momentum transfer *p*_i_ + *p*_f_ is followed by the thickness oscillations originating from the layered structure of the sample. The off‐specular scattering in Figure 4B appears as intensity bands perpendicular to the reflectivity line and crossing it at the thickness oscillation positions. The off‐specular scattering measured in the sample under the circular polarization conditions shows a strong scattering at 0.8 nm^−1^ < *Q* < 1.2 nm^−1^ (marked with the dashed box), which is absent in two other 2D maps measured in linear polarization and dark conditions. This intensity originates from the spin‐flip scattering of neutron spins on magnetic dipoles shown schematically in Figure 4A. In PNR measurements, the specular reflectivity part is determined by the magnetization components, which are parallel to the neutron polarization direction (Figure 3B), whereas the perpendicular component of the magnetization vector determines the spin‐flip as shown in the off‐specular scattering part in Figure 4B. Thus, specular reflection and off‐specular scattering provide complementary and complete information on the optically induced magnetization observed at MAPbBr_3_/Co interface.

In conclusion, we have presented experimental evidence of optically induced magnetization at perovskite/ferromagnet (MAPbBr_3_/Co) interface under photoexcitation by using the depth‐sensitive PNR method at room temperature. We observed optically induced magnetization in 7.5 nm of MAPbBr_3_ layer in the vicinity of ferromagnetic Co when orbital magnetic dipoles in MAPbBr_3_ are generated in the same direction under a circularly polarized photoexcitation. In contrast, no optically induced magnetization can be detected within the perovskite surface in the MAPbBr_3_/Co sample when the orbital magnetic dipoles are generated with opposite directions under a linearly polarized photoexcitation. This indicates that circularly polarized light‐generated spin states in the perovskite layer can directly interact with ferromagnet Co through electric‐magnetic coupling, leading to an optically induced magnetization. These studies represent an exciting discovery of the photoinduced room‐temperature ferromagnetism in metal halide perovskite in heterostructure design and have far‐reaching consequences, as it paves the way for the realization of new ferromagnetically tunable semiconductor materials through optical method for next‐generation spin‐related optoelectronics.

## 3 Experimental Section

##### Sample Preparation

The perovskite precursor solution was prepared by mixing the Br‐based solution and Ac‐based solution with the volume ratio of 1:9. The Br‐based solution was prepared by dissolving lead(II) bromide (PbBr_2_, 99.999%, metals basis, Alfa Aesar, 281 mg) and methylammonium bromide (MABr, 99.999%, 1‐Materials, 90 mg) into *N*,*N*‐dimethylformamide solvent (DMF, anhydrous, 99.8%, Sigma‐Aldrich, 1 mL). The Ac‐based solution was prepared by dissolving MABr (235 mg) and lead (II) acetate trihydrate (PbAc_2_∙3H_2_O, 99.999%, trace metals basis, Sigma‐Aldrich, 178 mg) into DMF (1 mL). Both solutions need to be freshly prepared before use. After mixing the two solutions, the obtained perovskite precursor solution was coated onto a precleaned Si wafer substrate (2 × 2 cm^2^) via a consecutive two‐step spin‐casting process at 500 rpm (250 rpm s^−1^) and 8000 rpm (4000 rpm s^−1^) for 7 and 60 s, respectively. The resulting films were then put in a petri dish for 5 min and annealed at 60 °C for 25 min to produce an MAPbBr_3_ thin film with good crystallization and an ultrasmooth surface over the entire substrate. Next, a 20 nm Co layer and 15 nm Au layer were thermally deposited under a vacuum of 5 × 10^−7^ Torr with deposition speed of 0.2 Å s^−1^.

##### Polarized Neutron Reflectometry

PNR experiments were performed on the Magnetism Reflectometer at the Spallation Neutron Source at Oak Ridge National Laboratory.^[^ ^30^ ^]^ Neutron scattering measurements were performed on a 2 × 2 cm^2^ surface samples. A neutron beam with a wavelength band of 2.6–8.6 Å with a high polarization of 99% to 98.5% was used. Measurements were performed at room temperature with an applied external magnetic field using a Bruker electromagnet with a maximum magnetic field of 1 T. Using the time‐of‐flight method, a collimated polychromatic beam of polarized neutrons with the wavelength band Δ*λ* impinges on the film at a grazing incidence angle *θ*, where it interacts with atomic nuclei and the spins of unpaired electrons (see Figure 2A). The reflected intensity is measured as a function of wave vector transfer, *Q *= 4*π*sin(*θ*)/*λ*, for two neutron polarizations *R*+ and *R−*, with the neutron spin parallel (+) or antiparallel (−) to the direction of the external field, *H*_ext_. To separate the nuclear from the magnetic scattering, the data are presented as the spin–asymmetry ratio, SA = (*R*+ − *R−*)/(*R*+ + *R−*) as depicted in Figures 2D and 3C. A value of SA = 0 designates no magnetic moment in the system. Being electrically neutral, spin‐polarized neutrons penetrate the entire multilayer structures and probe magnetic and structural composition of the film through the buried interfaces down to the substrate. A 488 nm continuous‐wave semiconductor laser was combined with a linear polarizer (GL10, Thorlabs) and quarter waveplate (AQWP05M‐600, Thorlabs) to generate switchable linearly and right‐hand circularly polarized laser beam excitation with identical intensity of 14 mW cm^−2^. Circularly polarized light creates magnetization direction parallel or antiparallel to the direction of the laser beam. During the PNR experiment, the external magnetic field is applied in plane of the film to maximize the sensitivity of the method. Thus, the magnetization induced in the sample is rotated to be aligned along the direction of the external magnetic field in the plane of the sample. Under these conditions, the handedness of the circularly polarized illumination does not affect the photoinduced magnetization in the case.

The fit to the PNR data was performed for two neutron spin‐states (R+ and R‐) simultaneously for the three sets of PNR measurements (circular, linear, and dark) and X‐ray reflectivity data. During the fit the structure parameters (layer thickness, scattering density, and the interfacial and surface roughness) were coupled for the three PNR data sets and also X‐ray reflectivity data, and only the magnetization depth profile was allowed to vary. In addition, in order to prevent local minimum fit converging, the mutation strategy is applied in the fitting procedure. The experimental error bars of R+, R−, and SA are calculated using the exact formula for propagation of error,^[^ ^31^ ^]^ which represent a direct statistical relationship between multiple variables and their standard deviations. The estimation of the physical parameters of a model was tested by varying the initial guesses within the physical limits of parameters in the model and comparing the resulting model parameters.

Statistical analysis procedure of the PNR data: 1) Preprocessing of data was performed using the data reduction software dedicated for the reduction of the experimental data at the Spallation Neutron Source (Oak Ridge National Laboratory).^[^ ^32^ ^]^ Experimental data were optimized for statistical accuracy so that the neutron scattering measurements were performed for sufficiently longer amount of time to obtain statistically significant results. 2) Data presentation: An error bar of one standard deviation is provided for the data wherever applicable. It is specified in the figure caption of relevant figures. 3) Sample size: Experimental results were reproduced on two samples, fabricated under identical conditions. The sample size was 2 × 2 cm^2^. 4) Statistical methods: Neutron scattering method is a well‐established statistical probe used to obtain information on structure and magnetization.^[^ ^33^ ^]^ 5) Software used for statistical analysis: a Licorne software developed for simulating and fitting neutron scattering data at grazing incidence was used.^[^ ^34^ ^]^

##### Other Characterizations

Absorption spectra were recorded by ultraviolet‐visible spectrometer (Lambda 35, from Perkin Elmer). The PL spectra were measured by fluorescence spectrometer (Horiba Fluorolog III) under the photoexcitation of 488 nm continuous‐wave laser. The magnetodielectric signals were measured by monitoring the capacitance as a function of the magnetic field strength. The capacitance was recorded by using an Agilent E4980A inductance, capacitance, and resistance (LCR) meter under an alternating electric field with a frequency of 1 kHz. The magnetodielectric effect is defined as$\;\mathrm{MFC}\;=\frac{C_{B}-C_{0}}{C_{0}}\;$and fitted by the Lorentzian function ($\mathrm{MFC}\;=\;\alpha \frac{B^{2}}{B^{2}+B_{0}^{2}}$), where *C*_B_ and *C*_0_ are the capacitance values in the situation with and without the magnetic field, respectively. X‐ray diffraction measurements were conducted on a Panalytical X'pert Materials Powder Diffractometer (MPD) Pro equipped with an X'Celerator solid‐state detector. For the X‐ray diffraction measurements, X‐rays were generated at 45 kV/40 mA, and the X‐ray beam wavelength was *λ* = 1.5418 Å (Cu *K*_*α*_ radiation). XRR data were measured using Panalytical X'pert Materials Research Diffractometer (MRD) Pro instrument with a Xe proportional counter. The voltage and current for both X‐ray generation were 45 kV and 40 mA, respectively. The measured reflectivity (*R*) versus *θ* curves were converted to *R* versus *Q*, where *Q* is equivalent to 4*π*sin(*θ*)/*λ* with *θ* being the incidence angle. *λ* was 1.5406 Å (Cu *K*_*α*1_ radiation). Magnetic hysteresis loop was taken by the Superconducting Quantum Interference Device measurement at room temperature.

## Conflict of Interest

The authors declare no conflict of interest.

## Author Contributions

The research was conceived and designed by B.H. The samples were prepared by M. W. and H. X. The optical characterizations were performed by M.W., H.X., I.N.I., T.W. and J.Q. The XRR data were collected and analyzed by J.K. The PNR measurements were carried out by M.W., H.X., H.A., and V.L. The PNR data were analyzed by V.L. M.W., V.L., and B.H. wrote the paper. All the authors contributed to the discussions.

## Supporting information



Supporting Information

## Data Availability

The data that support the findings of this study are available from the corresponding author upon reasonable request.
