# Supplementary material for: Optically Induced Static Magnetization in Metal Halide Perovskite for Spin‐Related Optoelectronics
Source: Adv Sci (Weinh). 2021 May 2;8(11):2004488. doi: 10.1002/advs.202004488 (PMC8188215; doi:10.1002/advs.202004488)
Supplement: Supplementary file 1 — Supporting Information [file ADVS-8-2004488-s001.pdf]

© 2021 Wiley-VCH GmbH

ADVANCED SCIENCE
Open Access

Supporting Information

for *Adv. Sci.,* DOI: 10.1002/advs.202004488

Optically Induced Static Magnetization in Metal Halide Perovskite for Spin-Related Optoelectronics

Miaosheng Wang[a], Hengxing Xu[a], Ting Wu[a], Haile Ambaye[b], Jiajun Qin[a], Jong Keum[b,c], Ilia N. Ivanov[c,d], Valeria Lauter[b,], Bin Hu[a,*]*

Supporting Information

Optically Induced Static Magnetization in Metal Halide Perovskite for Spin-Related Optoelectronics

Miaosheng Wang[a], Hengxing Xu[a], Ting Wu[a], Haile Ambaye[b], Jiajun Qin[a], Jong Keum[b,c], Ilia N. Ivanov[c,d], Valeria Lauter[b,*], Bin Hu[a,*]

[a] *Joint Institute for Advanced Materials, Department of Materials Science and Engineering, University of Tennessee, Knoxville, TN 37996, USA*

[b] *Neutron Scattering Division, Neutron Sciences Directorate, Oak Ridge National Laboratory, Oak Ridge, TN 37831, United States*

[c] *Center for Nanophase Materials Science and Chemical and Engineering Materials Division, Oak Ridge National Laboratory, Oak Ridge, TN 37831, United States*

[d] *Chemical and Engineering Materials Division, Oak Ridge National Laboratory, Oak Ridge, TN 37831, United States*

Corresponding authors:

Valeria Lauter: lauterv@ornl.gov

Bin Hu: bhu@utk.edu

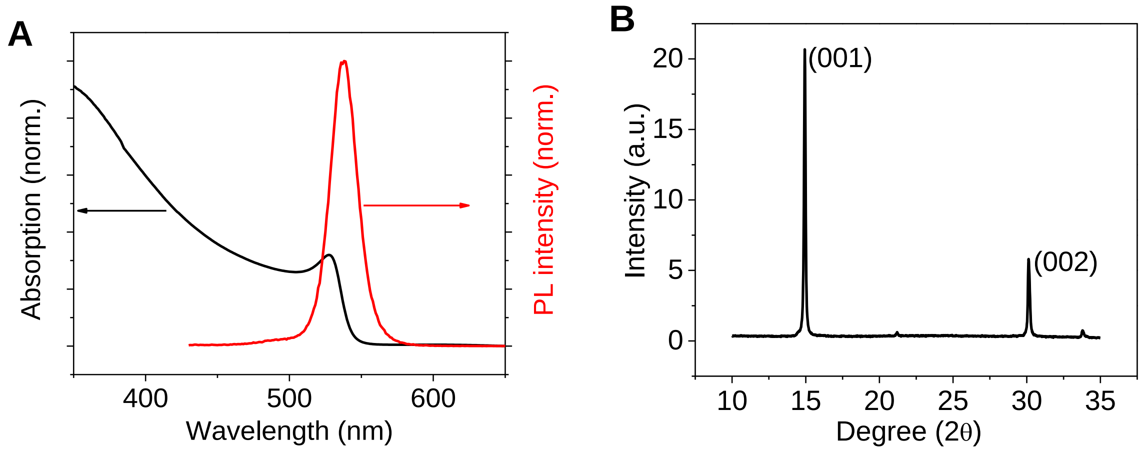

Figure S1. (A), Absorption and PL spectra of the $MAPbBr_3$ thin film. **(B)**, Out-of-plane θ-2θ X-ray diffraction scan for $MAPbBr_3$ thin film.

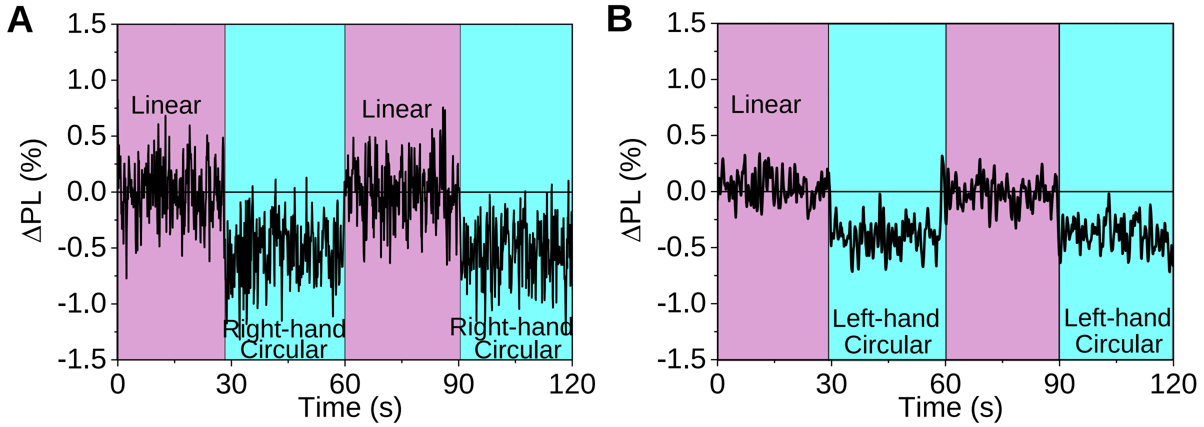

Figure S2. PL change caused by switching the photoexcitation from linear to **(A)** right-hand and **(B)** left-hand circular photoexcitation. The orbital magnetic dipoles are formed in spin-polarized excited states under circularly polarized photoexcitation.

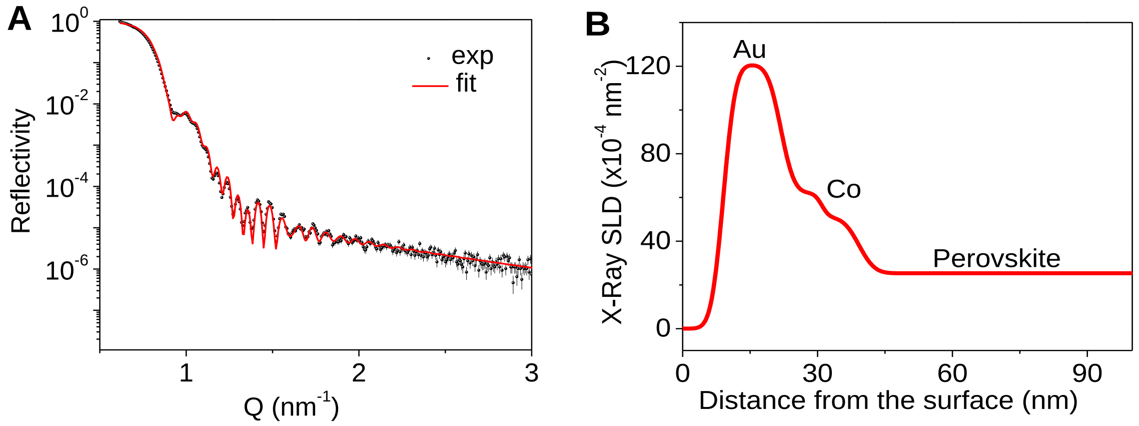

Figure S3. (**A)**, X-ray reflectivity (XRR) data for the Si/$MAPbBr_3$/Co/Au sample. The black open circles show the experimental data, and the fit to the data is shown in red. (**B)**, Chemical depth profile for the Si/$MAPbBr_3$/Co/Au sample obtained after the fit to the XRR data. The fit to the data showed that the density of the Co layer is not uniform. Instead, it forms two sublayers, Co_1 and Co_2, which exhibit lower and higher SLD values. This might be caused by the density difference during the deposition process of the Co layer on the surface of the perovskite.

Table S1. Fit parameters of the XRR data for Si/$MAPbBr_3$/Co/Au sample

Layers	Thickness, nm	SLD, $\times 10^{-4}$ nm^{-2}	Roughness, nm
Si	inf	20.1	2.14
SiO_2	1.375	18.9	2.33
Perovskite	65.86	25.3	1.29
Co_1	8.28	51.1	2.75
Co_2	9.09	61.9	0.02
Au	12.79	120.7	0.02

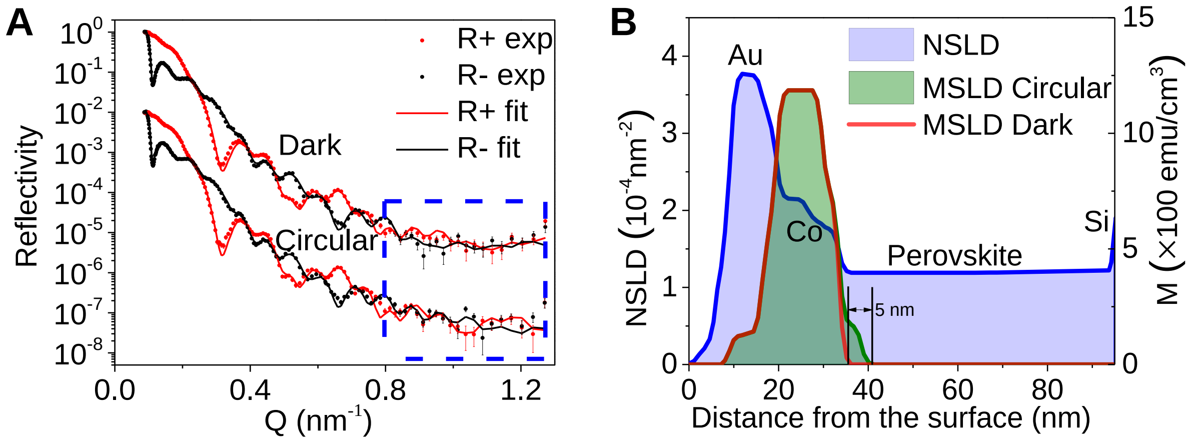

Figure S4. (A), Polarized neutron reflectivity plots of the first sample (N1), as a function of scattering vector magnitude Q, for a film measured in dark (top) and under circularly polarized photoexcitation (bottom). The experimental data are shown as black points with fits shown as overlying lines; **(B)**, The full neutron nuclear and magnetic scattering length density profiles (NSLD and MSLD) obtained from the fit to the reflectivity.

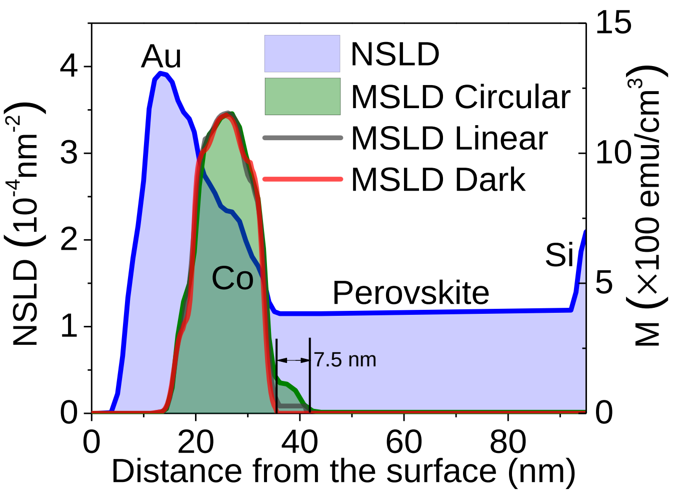

Figure S5. The full neutron nuclear and magnetic scattering length density profiles (NSLD and MSLD) obtained from the fit to the reflectivity of the second sample (N2).

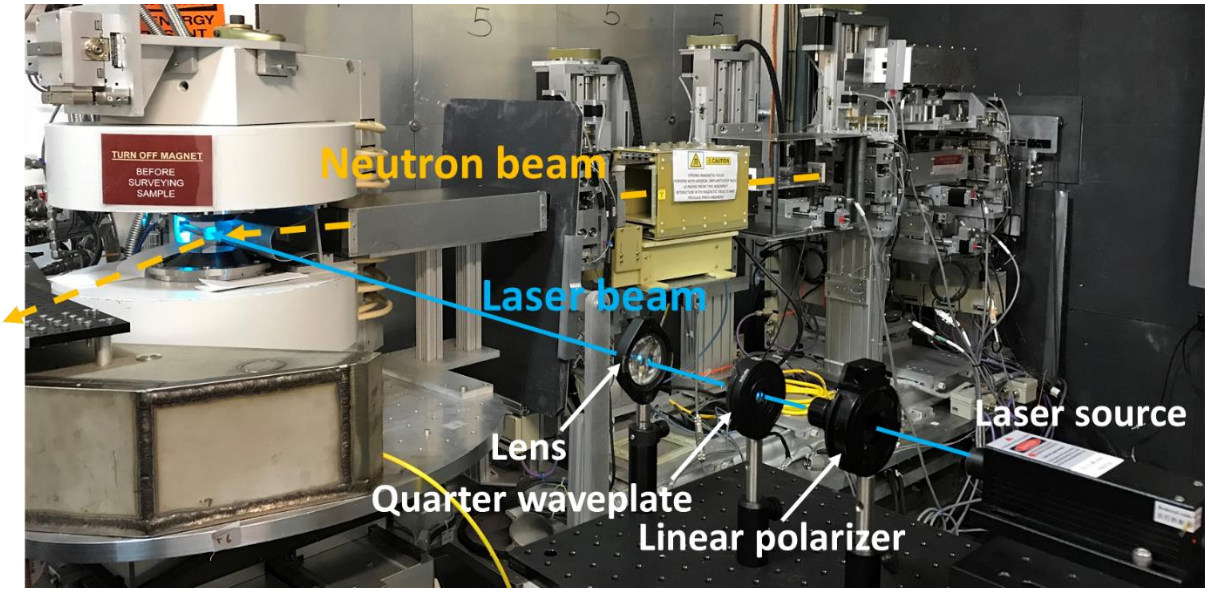

Figure S6. Experimental setup at the Magnetism Reflectometer for *in situ* photoexcitation with linear and circular polarization. The photoexcitation incident angle is ~10 degrees to the normal of the sample plane. The neutron beam (shown by the yellow dashed line) contains spin parallel (+) or antiparallel (−) neutrons with respect to the direction of the external field. The optical setup contains the laser source, linear polarizer, quarter waveplate, and lens and is used to provide the circularly and linearly polarized light (shown with blue arrow) with the same

intensity. The PNR measurements were performed with *in situ* photoexcitation and dark conditions.
